# Dietary restriction with and without caloric restriction for healthy aging

**DOI:** 10.12688/f1000research.7136.1

**Published:** 2016-01-29

**Authors:** Changhan Lee, Valter Longo

**Affiliations:** 1 Davis School of Gerontology, University of Southern California, Los Angeles, CA, USA; 2 IFOM - FIRC Institute of Molecular Oncology Foundation, Milan, Italy

**Keywords:** Caloric restriction, mechanisms of aging, Dietary restriction, aging, healthspan

## Abstract

Caloric restriction is the most effective and reproducible dietary intervention known to regulate aging and increase the healthy lifespan in various model organisms, ranging from the unicellular yeast to worms, flies, rodents, and primates. However, caloric restriction, which in most cases entails a 20–40% reduction of food consumption relative to normal intake, is a severe intervention that results in both beneficial and detrimental effects. Specific types of chronic, intermittent, or periodic dietary restrictions without chronic caloric restriction have instead the potential to provide a significant healthspan increase while minimizing adverse effects. Improved periodic or targeted dietary restriction regimens that uncouple the challenge of food deprivation from the beneficial effects will allow a safe intervention feasible for a major portion of the population. Here we focus on healthspan interventions that are not chronic or do not require calorie restriction.

## Introduction

Aging is the leading risk factor for many among the major diseases and a key factor in the overall decline of physical and mental performance. Interventions that slow down the aging process can delay or prevent multiple chronic diseases and improve productivity and quality of life in older ages. For clarity, here, we use caloric restriction (CR) to refer to a dietary intervention with an overall 20–40% reduction of total caloric intake, and dietary restriction to represent a broader scope of dietary interventions that encompass those with specific macronutrient and feeding pattern restrictions. CR is the most studied and reproducible non-genetic intervention known to extend healthspan and/or lifespan in organisms, ranging from unicellular yeast to monkeys. It started with a simple experiment where a reduction in dietary intake (i.e. caloric restriction) extended the lifespan of rats ^1^, providing a foundation to experimentally study the relationship between nutrition and the biology of aging. Here we discuss more recent discoveries that have advanced our knowledge of the effects of less invasive and restrictive dietary interventions in aging and healthspan.

## Caloric restriction and the conserved mechanisms of aging

In their seminal report in 1935, Crowell and McCay demonstrated that simply reducing caloric intake without causing malnutrition nearly doubled the lifespan of rats ^1^, providing an experimental model to begin to demonstrate that aging can be slowed down. Nearly half a century later, Walford and Weindruch reported that “adult-initiated” caloric restriction started at 12 months of age not only increased lifespan but also reduced the incidence of spontaneous cancer by more than 50% in rats ^2, 3^. Several decades later, the effect of caloric restriction on healthspan and lifespan has been confirmed in model organisms ranging from unicellular yeast to worms, flies, rodents, and primates ^4^, suggesting a highly conserved effect which may involve common genes. Although the molecular mechanisms that mediate the effect of caloric restriction are still being investigated and debated, there is more widespread acceptance of the hypothesis that caloric restriction and lifespan extension involves the down-regulation of insulin and insulin-like signalling (IIS) ^5^, as well as of the amino signalling target of rapamycin (TOR)-S6 kinase pathway ^6, 7^, and the glucose signalling Ras-protein kinase A (PKA) pathway ^6, 8, 9^.

Because an in depth discussion of the anti- and pro-aging pathways conserved in model organisms has been covered elsewhere, here we will only mention the most relevant ones ^10– 12^. In yeast, down-regulation of (a) the amino acid-sensing TOR and the ribosomal protein S6 kinase (S6K) ortholog Sch9 pathway ^6^, and (b) the Ras-AC-PKA pathway ^13^ are key changes mediating part of the effects of caloric restriction on chronological lifespan, the measurement of cellular survival under non-dividing conditions. In contrast, elevated activity of sirtuin ( *SIR2*) has been described as a key change in the extension of replicative lifespan, measured by counting the number of buds generated by an individual mother cell ^14, 15^. In worms, the lifespan extension caused by the inactivation of IIS, or by different forms of caloric restriction, requires Forkhead FoxO transcription factor daf-16 ^16^. In flies, the IIS pathway is involved in the effects of caloric restriction ^17^ and, although dFoxo is not required for its longevity effect, its activity can affect the response to caloric restriction ^18^. In rodents, growth hormone (GH) and IGF-1 levels are reduced following caloric restriction ^19^, but the link between dietary restriction, GH and aging is still being investigated, with focus on the genes and pathways regulating longevity in the simple organisms described above. The long-lived GH receptor knock-out (GHRKO) mice, which are resistant to GH, do not exhibit further lifespan extension or health benefits by caloric restriction ^20, 21^, but the long-lived GH-deficient Ames mice do ^22^, suggesting a complex involvement of the GH/IGF-1 axis and periphery pathways in the response to caloric restriction.

Much has been learned about caloric restriction and aging from model organisms, but the ultimate question that lingers is the relevance of these models to human lifespan and healthspan. The rhesus monkeys are the closest model organism to humans in which caloric restriction has been experimentally tested in a controlled environment. Two notable studies performed by independent programs, the National Institute on Aging (NIA) Intramural Research Program and the Wisconsin National Primate Research Center (WNPRC), subjected male and female rhesus monkeys to 30% caloric restriction from levels of baseline caloric intake. The NIA reported no improvement in lifespan but observed a positive trend for the delay of age-related diseases (i.e. healthspan) ^23^, whereas WNPRC reported significant improvement in both lifespan and healthspan ^4, 24^. The discrepancies are largely attributed to the different dietary composition and heterogenic genetic background ^4, 23^, which have been shown to be a significant factor in rodents ^25, 26^. This underscores the importance of diet composition and genetic background and their compatibility when applying caloric restriction to humans. Nonetheless, several studies provide evidence supporting beneficial health effects of caloric restriction for humans. A notable NIH-sponsored controlled randomized study on non-obese individuals, CALERIE (Comprehensive Assessment of the Long-term Effects of Reducing Intake of Energy), recently reported that a two year 25% caloric restriction is feasible for humans and provides health benefits, such as reduced inflammatory markers and cardiometabolic risk factors ^27– 29^. However, caloric restriction was associated with reduced bone mineral density and exercise was recommended to offset such adverse effects ^30^. Notably, CALERIE was conducted in three independent centers and involved 218 overweight participants, suggesting that caloric restriction can be beneficial even in a very genetically heterogeneous group ^31^. However, considering the results in monkeys, much longer and larger studies will be needed to know what the effects of CR on human healthspan.

Among the cellular alterations most closely associated with both caloric restriction and longevity mutations is the resistance to multiple stressors, which in most cases includes resistance to oxidative stress. The ability of caloric restriction to prevent the damage caused by exogenous toxins is likely to be associated with the protection, repair and replacement effects that prevent the age-dependent dysfunction caused by endogenous processes and toxic molecules ^32^. An alternative hypothesis suggests that caloric restriction acts as a mild stressor that promotes hormesis, which refers to the beneficial effects resulting from the cellular responses to mild, repeated stress ^33^. Stress resistance should also be considered an important criterion for the successful development of caloric restriction-mimetic dietary and pharmacological interventions.

## Dietary restriction: macromolecular restriction without caloric restriction

The definition of dietary restriction has been expanded from an alternative description of caloric restriction to also encompass a broader scope of interventions, including short-term starvation, periodic fasting, fasting-mimetic diets, intermittent fasting, normo-caloric diets with planned deficiencies (in particular macronutrients: proteins, carbohydrates, etc.), and time-restricted feeding. Most of these relatively novel interventions are reported to have beneficial effects on overall health and in some cases longevity. Fasting is an extreme dietary intervention describing either a complete lack of food intake or a 60% or higher food restriction. Intermittent fasting refers to practicing this intervention every other day whereas periodic fasting refers to severe restriction for two or more days periodically (every two weeks, month, etc.). Caloric restriction and fasting share similar but often distinct effects on a number of biomarkers (e.g. reduced glucose, and insulin levels) suggesting that partially overlapping mechanisms are involved ^19^. Both intermittent and periodic fasting can increase lifespan, even when there is little or no overall decrease in calorie intake ^34, 50^.

### Macronutrient restriction

In addition to periodic or intermittent fasting-based strategies as alternatives to caloric restriction, the restriction of specific macronutrients (or macronutrient restriction) without the restriction of calories is among the most promising interventions that have emerged to promote healthy aging in humans. Among the different types of macronutrient restriction, reduced intake of proteins and amino acids is the most effective pro-longevity intervention ^35, 36^. Simply reducing protein intake can deliver an equally potent impact on lifespan as dietary restriction in multiple model organisms ^35^. A recent analysis of the National Health and Nutrition Examination Survey (NHANES) showed that low protein intake was associated with reduced overall mortality for those under 65 years of age ^37^. Also, a high-carbohydrate, low-protein diet resulted in longer lifespan and improved cardiometabolic health, despite increased food intake and body fat ^38, 39^. Furthermore, the restriction of a single essential amino acid in a normal diet increased lifespan and stress resistance ^40– 44^. In flies, adding back essential amino acids to the caloric restriction diet decreased lifespan to that of the normally fed group ^36^. Laboratory rodents fed a methionine-restricted diet displayed an extended lifespan with decreased age-dependent diseases and increased resistance to oxidative stress, in part due to increased antioxidant capacity ^44– 48^. A tryptophan-restricted diet also provided longevity and reduced age-dependent deterioration ^42, 43, 49^ but has mainly been explored for neurological benefits, due to its role in serotonin synthesis. A fasting-mimicking diet, consisting of very low calorie and protein that leads to similar physiological response to fasting, including reduced levels of glucose and IGF-1 and increased levels of ketone bodies IGFBP-1, enhanced healthspan and rejuvenated the hematopoietic system while promoting adult neurogenesis ^50^. Further studies on carbohydrate and fat restriction are needed to determine their role in dietary restriction.

### Restriction of feeding time

Feeding schedule has also been shown to have a significant impact on health and survival. In flies, time-restricted feeding (limited to 12 daytime hours every day) had profound effects on neural, peripheral, and cardiovascular physiology and improved sleep, body weight maintenance, and delayed signs of cardiac aging, under unchanged caloric intake and activity ^53^. When mice were given access to food for only 8–9 hours during the active phase of the day, metabolic diseases induced by a high-fat, high-fructose, and high-sucrose diet, were reduced without lowering caloric intake ^51^. The benefits of time-restricted feeding against such obesogenic diets were proportional to the duration of the fasting each day ^52^. *Ad lib* feeding during the weekend did not interfere with the protective effects of time-restricted feeding ^52^. Notably, the restricted feeding pattern reversed the progression of pre-existing obesity and type II diabetes, suggesting it has the potential to be a clinically relevant and feasible dietary intervention, useful to prevent and treat obesity and metabolic disorders ^52^. Considering that key metabolic factors, such as 5' AMP-activated protein kinase (AMPK), sirtuins, and protein kinase B (AKT), are regulated by an interplay of circadian rhythm and feeding time ^54, 55^, dietary schedules should be more carefully studied in the context of dietary restriction.

## Conclusion

Dietary interventions that extend healthspan and lifespan have evolved and have become much better characterized since the original caloric restriction experiments performed by McCay in 1935. We now understand that its effects on aging are not simply the result of the reduced amount of calories consumed, but are also determined by diet composition, and can be achieved by periodic interventions which do not require an overall reduction in calorie intake and which can be achieved without a complete lack of food intake during the periodic fasting cycles. Further studies are important to identify even less invasive and more effective dietary interventions that will cause coordinated and beneficial effects on healthspan.
